# HPV-associated squamous cell carcinoma in situ arising from urethral caruncle: A case of the unusual occurrence in the common urogenital lesion

**DOI:** 10.1016/j.ijscr.2025.111881

**Published:** 2025-08-29

**Authors:** Jungsup Byun, Nora Jee-Young Park, An Na Seo, Ghilsuk Yoon

**Affiliations:** a Department of Pathology, School of Medicine, Kyungpook National University, Daegu, Republic of Korea; b Department of Pathology, School of Medicine, Kyungpook National University, Kyungpook National University Chilgok Hospital, Daegu, Republic of Korea; c Department of Pathology, Daegu Fatima Hospital, Daegu, Republic of Korea

**Keywords:** Urethral caruncle, Neoplasm, Squamous cell carcinoma, Human papillomavirus, Case report

## Abstract

**Introduction and importance:**

Urethral caruncle is one of the most prevalent urethral inflammatory disease in postmenopausal females. Although urethral caruncle is neither neoplastic nor preneoplastic, there have been rare reports of malignant neoplasm arising from them. Furthermore, none of them revealed association between urethral caruncle lesion and HPV (human papilloma virus) infection.

**Case presentation:**

We herein report a case of an 80-year-old female with squamous cell carcinoma in situ arising from the urethral caruncle, which was found to be associated with HPV infection. The 1.5 cm-sized mass located at posterior aspect of urethral meatus, was clinically suspected urethral caruncle.

**Clinical discussion:**

After caruncle excision was performed, histopathological result demonstrates severe squamous dysplasia with koilocytic atypia, involving the whole thickness of squamous epithelium. No invasive tumour cell was identified, consistent with squamous cell carcinoma in situ. The lesion showed block positivity in p16 immunohistochemical staining, which is the surrogate marker for the cytopathic incorporation of human papilloma virus. In the following molecular study, viral expression of HPV was confirmed by real time PCR with detection of high-risk HPV subtype 39.

**Conclusion:**

Squamous cell carcinoma originating from a urethral caruncle is an exceptionally uncommon occurrence. A comprehensive review on the English-written literature yielded only three previously documented case reports.

## Highlights

• Urethral caruncles are conventionally understood as benign, non-neoplastic lesions.
• Squamous cell carcinoma arising from a urethral caruncle is exceptionally rare.
• This is the first documented case associated with high-risk HPV infection, suggesting a novel viral oncogenic pathway in this lesion.

## 1 Introduction

Urethral caruncle is a benign polypoid mass located near the posterior lip of urethral meatus. It is predominantly found in postmenopausal women, and usually presents as pink fleshy and/or friable nodule with the size of 0.2 to 3 cm (in average, 0.95) [1,2]. Macroscopically, they appear as nodular or pedunculated erythematous lesions prone to bleeding. The lesion is considered to be resulted from recurrent local trauma or inflammation, in combination with hypoestrogenic status of postmenopausal women [3]. Histologically, these lesions comprise a connective tissue core covered by urothelial or squamous epithelium.

While a definitive link to malignancy, urologic disorders, or systemic disease has not been confirmed, urethral caruncle often presents a challenging clinical differential diagnosis that includes malignancy. In contrast, some cases that were clinically considered just a caruncle unexpectedly reveal malignancy. In one of the most comprehensive study examining urethral neoplasms and caruncles to date, histopathological analysis of 376 excised urethral lesions, initially diagnosed as benign caruncles, revealed malignancy in 9 cases (2.4 %) [4].

Squamous cell carcinoma developing from a urethral caruncle is an exceptionally rare occurrence. Herein, we present a case of Human Papillomavirus (HPV)-associated squamous cell carcinoma (SqCC) in situ arising from the urethral caruncle, following the case reporting guideline of SCARE criteria [5].

## 2 Case report

A 80-year-old female presented with vaginal bleeding and urethral mass for over several weeks. The patient had medical history of palliative chemoradiation therapy for pancreatic cancer with peritoneal metastasis. There is no history of abnormal cervicovaginal Pap smear test, of which the latest test was a year ago. The patient's history revealed no discernible risk factors associated with urothelial cell carcinoma or cervical squamous cell carcinoma, such as tobacco use, occupational exposure to carcinogens, or problematic sexual behavior.

**Fig. 1:**
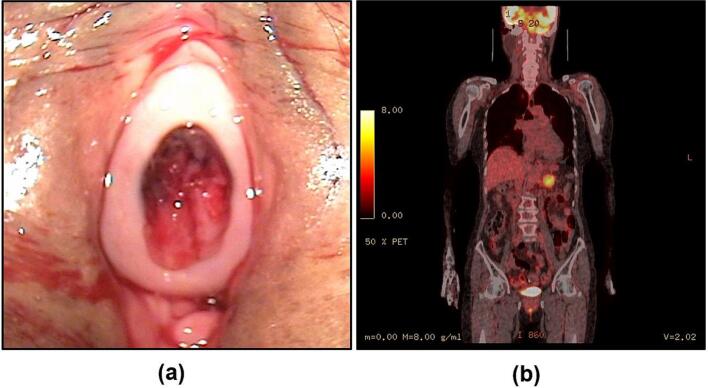
(a) Urethral caruncle: a blood‑tinged sessile polypoid mass located posterior aspect of urethral meatus. The mass was friable and clearly the source of bleeding. (b) PET-CT scan previously reported no specific comment on the urethral region: Retrospective review after the pathologic confirmative diagnosis revealed suspicious high [18]FDG uptake in the region.

Gynecological speculum examination revealed no bleeding focus along the vaginal and intrauterine cavity, but a 1.5 cm-sized blood‑tinged sessile polypoid mass (Fig. 1.a), located posterior aspect of urethral meatus, consistent with urethral caruncle. The mass was friable and clearly the source of bleeding. A caruncle excision was performed under general anesthesia without complication and the specimen was sent for pathology.

**Fig. 2:**
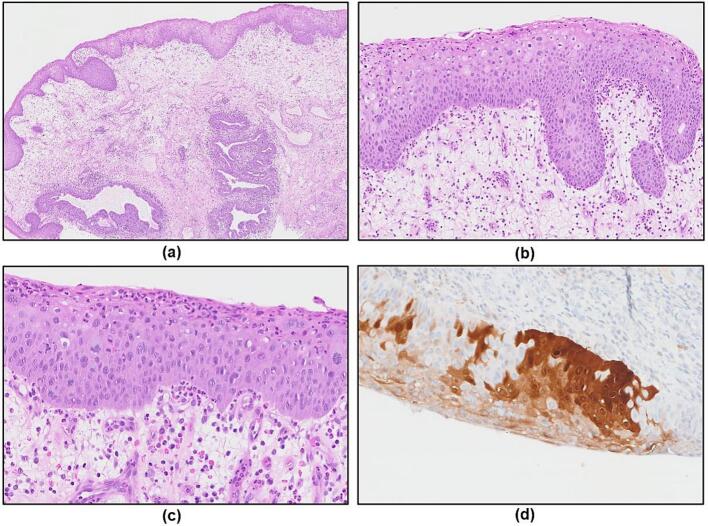
(a) Urethral caruncle at low magnification, demonstrating a polypoid lesion lined by hyperplastic urothelial and squamous epithelium. The underlying stroma demonstrated edema and dilated blood vessels, with occasional invaginated rounded nests of urothelium. (b) The lesion with full-thickness nuclear abnormality, consistent with high-grade squamous intraepithelial lesion (HSIL). (c) Focal areas with marked nuclear atypia, manifesting as nucleomegaly and occasional multinucleation. Brisk mitotic activity and dispersed atypical mitotic figures are seen. (d) The immunostaining of p16 shows a strong diffuse block positivity.

Histopathologic examination demonstrates a typical urethral caruncle showing a polypoid lesion lined by hyperplastic urothelial and squamous epithelium. Stromal edema and ectasia of blood vessels with RBC extravasaion and inflammatory infiltrates were present. Rounded nests of urothelium arranged in single or multiple invaginated architecture were seen with overlying squamous metaplasia (Fig. 2.a). Among the squamous cell linings, significant nuclear abnormalities throughout the full thickness were noted. These abnormalities were characterized by hyperchromasia, coarse or vesicular chromatin, and irregular nuclear membranes. Additionally, an increased N:C ratio and brisk mitotic activity were observed (Fig. 2.b). Focal areas exhibited marked nuclear atypia, manifesting as nucleomegaly and occasional multinucleation. Atypical mitotic figures were dispersed throughout the epithelium, notably present even in the superficial layers (Fig. 2.c). However, invasive foci were not identified. These features collectively indicate a high-grade squamous intraepithelial malignant process, consistent with the diagnosis of SqCC in situ. The immunostaining of p16 shows a strong diffuse block positivity (Fig. 2.d), which is an acceptable surrogate marker of HPV association. In the following molecular study, the presence of HPV DNA was confirmed by real time PCR with detection of high-risk HPV subtype 39.

Following the unexpected confirmation of malignancy in the urethral caruncle through pathological diagnosis, a retrospective review of the previously reported unremarkable PET-CT scan was conducted. This re-examination revealed a previously unnoticed area of suspiciously high FDG uptake in the urethral region (Fig. 1.b). The presence of this metabolically active focus, initially overlooked or neglected, correlates with the subsequent histopathological findings of malignancy.

## 3 Discussion

Urethral caruncles represent the most prevalent urethral pathology in females, typically manifesting as inflammatory nodules at the posterior lip of the external meatus, particularly in postmenopausal women. The pathogenesis is not well understood, but it is thought that a distal segment of the urethral mucosa prolapses to form the caruncle. According to the histopathologic study of urethral caruncle examining 41 cases, the largest study so far, urethral caruncles exhibit a hyperplastic epithelial lining of benign urothelium and squamous epithelium, with frequent urothelial invaginations into the stroma, often showing squamous metaplasia and cystic spaces [2]. The stromal compartment demonstrated edema, vascular dilation, inflammatory infiltrates, and fibrosis, with all cases showing diffuse chronic inflammation. Notably, significant cytologic atypia and atypical mitoses were absent in the epithelial component across all examined cases [2]. Traditionally, caruncles are considered benign and not associated with neoplastic risk [2].

In the present case, the patient's urethral caruncle exhibited only a few atypical clinical features, namely persistent bleeding and a relatively large size. While macroscopically suggestive of a usual caruncle, histopathological examination revealed severe squamous dysplasia throughout the epithelium, with focal areas of marked nuclear atypia of nucleomegaly, multinucleation, and atypical mitotic figures. Consequently, the diagnosis of intraepithelial SqCC arising from a urethral caruncle was established. And further immunohistochemical and molecular study confirmed its association with high-risk HPV infection.

This finding challenges the conventional understanding of urethral caruncles as non-neoplastic lesions. While chronic inflammation by repetitive irritation such as indwelling catheter or stones is recognized as a risk factor for SqCC in patients with bladder cancer [3], its role in urethral caruncle malignant transformation remains uncertain.

A comprehensive review of English literature revealed only three previously reported cases of intraepithelial SqCC arising from urethral caruncles. Marshall et al., in his analysis of 376 cases of urethral caruncle resection, reported one case of intraepithelial SqCC coexisting with “benign appearing” urethral caruncles [4]. Kaneko et al. and Shim et al. described the other cases of SqCC in situ arising from a urethral caruncle [6,7]. To our best knowledge, the present case represents the fourth documented instance of this rare entity. The literature review was expanded to encompass a broader spectrum of neoplasms, extending beyond SqCC specifically arising from urethral caruncles. (Table I). In addition to the three previously mentioned cases, nine other instances of neoplasms arising from or associated with histologically confirmed urethral caruncles were identified, including squamous cell papilloma, urothelial cell carcinoma, and MALT-type lymphoma, etc. None of these cases were found to be associated with HPV infection. Therefore, our case is the first published case of HPV associated urethral caruncle lesion.

**Table I:** Literature review of published cases with pathologic diagnosis of neoplasm arising from or associated with urethral caruncle.

Reference	Year	Age (Y)	Sex	Size (mm)	Presenting symptoms	Diagnosis of the neoplasm
Marshall et al. [4]	1960	NA	NA	NA	NA	Squamous cell carcinoma in situ
Kaneko et al. [6]	2011	62	F	5^a^	Vaginal bleeding	Squamous cell carcinoma in situ
Shim et al. [7]	2013	52	F	17^a^	Palpable mass and pain	Squamous cell carcinoma in situ
Gustafson et al. [8]	2014	43	F	NA	Dysuria and hematuria	Squamous cell papilloma
Çelik et al. [9]	2018	18	M	5^a^	Dysuria	Squamous cell papilloma
Sengupta et al. [10]	2021	22	F	20 × 15	Voiding difficulty and hematuria	Squamous cell papilloma
Omar et al. [11]	2007	57	F	NA	Vaginal bleeding	Urothelial cell carcinoma
Safadi et al. [12]	2017	52	F	30^a^	Vaginal bleeding	Primary malignant melanoma of urethra
Bansal et al. [13]	2018	50	F	10 × 10	Dysuria and vaginal bleeding	Primary malignant melanoma of urethra
Cimentepe et al. [14]	2005	57	F	30 × 30	Hematuria	Adenocarcinoma
Chen et al. [15]	2014	63	F	50 × 45	Hematuria	MALT-type lymphoma
Palmisano et al. [16]	2018	84	F	40^a^	Vaginal bleeding and dysuria	Mantle cell lymphoma

M: Male, F: Female, NA: Not available.

a Size measured in the greatest dimension.

Given its rarity, no standardized treatment protocol exists for intraepithelial carcinoma arising from urethral caruncles. Primary urethral carcinoma in females is uncommon, accounting for 0.02 % of all female cancers and 5 % of urologic malignancies [3]. Distal urethral carcinomas generally present at an early stage, with local excision alone yielding cure rates between 70 % and 90 % [3]. However, meticulous follow-up is essential in this case.

It is noteworthy that certain neoplasms, including primary malignant melanoma, non-Hodgkin lymphoma, primary adenocarcinoma, can mimic urethral caruncles [14,17,18]. Urethral carcinoma or other malignancy masquerading as a urethral caruncle, while rare, underscores the importance of maintaining a high index of suspicion in atypical presentations.

## 4 Conclusions

The occurrence of malignancy arising from a urethral caruncle, or urethral carcinoma mimicking a caruncle, is exceedingly rare. Our case is the fourth published instance of intraepithelial SqCC originating from urethral caruncle, and the first to be associated with high-risk HPV infection. The potential pathogenetic relationship between high-risk HPV types and urethral squamous dysplastic lesions warrants further investigation. In addition, this case emphasizes the need for cautious diagnostic approach when encountering atypical presentations of urethral caruncles.

## Author contribution

J.B., G.Y.: concept, design, final approval, draft writing; J.B., N.J., A.N.: analysis, interpretation, draft writing; N.J., A.N., G.Y.: critical revision, final approval.

## Consent

Written informed consent was obtained from the patient for publication and any accompanying images. A copy of the written consent is available for review by the Editor-in-Chief of this journal on request.

## Ethical approval

This study was approved by the Institutional Ethics Committee (please specify name of the Institution: Kyungpook National University Chilgok Hospital) (approval number/protocol number KNUCH 2024-11-008-002). The research was conducted ethically, with all study procedures being performed in accordance with the requirements of the World Medical Association's Declaration of Helsinki. Written informed consent was obtained from each participant/patient for study participation and data publication.

## Guarantor

Jungsup Byun, Ghilsuk Yoon.

## Research registration number

KNUCH 2024-11-008-002.

## Funding

We have no disclosure of funding for this work.

## Conflict of interest statement

The authors declare no conflict of interest.
